# Nalbuphine’s Hemodynamic Impact in Ear, Nose, and Throat (ENT) Surgeries: A Comprehensive Review

**DOI:** 10.7759/cureus.52755

**Published:** 2024-01-22

**Authors:** Dhruv Shah, Jayashree Sen

**Affiliations:** 1 Anesthesia, Jawaharlal Nehru Medical College, Datta Meghe Institute of Higher Education and Research, Wardha, IND

**Keywords:** clinical outcomes, anesthesia protocols, opioid receptors, ent surgeries, hemodynamic impact, nalbuphine

## Abstract

Nalbuphine, a semi-synthetic opioid, has gained attention for its analgesic properties, but its specific impact on hemodynamics in ear, nose, and throat (ENT) surgeries remains a subject of exploration. This comprehensive review aims to systematically analyze existing literature to understand the nuanced hemodynamic effects of nalbuphine during ENT procedures. Nalbuphine demonstrates promise as an analgesic agent in ENT surgeries with generally stable hemodynamic profiles. However, the variability in study designs and outcomes necessitates a cautious interpretation. The review underscores the need for standardized protocols and further research to elucidate patient-specific considerations, ensuring optimal utilization of nalbuphine in enhancing overall perioperative care for ENT patients.

## Introduction and background

Nalbuphine, a semi-synthetic mixed agonist/antagonist opioid modulator of the phenanthrene or morphinan series, has garnered attention in perioperative care due to its distinctive pharmacological profile [1]. Originally introduced as an analgesic in the late 1970s, Nalbuphine exhibits a nuanced opioid receptor activity, simultaneously acting as an agonist on kappa receptors and an antagonist on mu receptors. This unique dual mechanism sets it apart from conventional opioids, making it an intriguing and differentiated choice for pain management [2].

The field of ear, nose, and throat (ENT) surgeries poses unique challenges, with procedures ranging from routine tonsillectomies to intricate rhinoplasties and functional endoscopic sinus (FES) surgeries [3-5]. Particularly in these cases, achieving controlled hypotension is often imperative. Controlled hypotension involves deliberately reducing blood pressure to enhance surgical visibility and minimize blood loss, demanding a delicate balance in cardiovascular parameters. The proximity of vital structures, intricate vascular anatomy, and the potential for bleeding make vigilant control over blood pressure and heart rate essential to mitigate complications and optimize patient outcomes [4]. It is noteworthy that procedures such as routine tonsillectomies, rhinoplasties, and FES surgeries may specifically demand hypotensive anesthesia [3-5].

The primary objective of this comprehensive review is to explore and synthesize existing literature on the hemodynamic impact of nalbuphine, specifically in the context of ENT surgeries. By delving into the pharmacology of nalbuphine and its unique receptor interactions, this review aims to elucidate its influence on critical cardiovascular parameters. Additionally, we will critically evaluate clinical studies and evidence, shedding light on the safety profile, adverse effects, and potential benefits of nalbuphine in the context of ENT surgical interventions. Through this synthesis, we hope to provide clinicians and researchers with a nuanced understanding of nalbuphine's role in optimizing hemodynamic stability during ENT procedures and identify avenues for future research in this domain.

## Review

Hemodynamic stability in ENT surgeries

Importance of Hemodynamic Stability

Controlled hypotension: Induced hypotension is used in ENT surgeries to enhance surgical visibility and minimize blood loss. However, it is imperative to uphold hemodynamic stability throughout the intraoperative period. A study that compared the utilization of dexmedetomidine and esmolol revealed that dexmedetomidine, functioning as an alpha-2 adrenergic agonist, exhibited superior efficacy in maintaining hemodynamic stability, particularly noted during FES surgery [5].

Prevention of hemodynamic instability: The occurrence of postoperative hemodynamic instability is influenced by various factors, including patient-specific elements, anesthesia-related considerations, and aspects intrinsic to the surgical procedure [6]. Identifying and proactively addressing these risk factors is pivotal in mitigating hemodynamic instability and improving overall patient outcomes.

Monitoring hemodynamic parameters: Rigorous monitoring of hemodynamic parameters, such as blood pressure, heart rate, and oxygen saturation, is indispensable during ENT surgeries. This meticulous observation ensures patient safety and facilitates the early detection of any deviations in hemodynamic status [5]. Such vigilant monitoring is crucial for timely interventions and contributes significantly to ENT procedures' overall success and safety.

Managing complications: Hemodynamic instability poses a potential risk for complications during and after ENT surgeries. The early identification and effective management of hemodynamic instability play a crucial role in enhancing patient outcomes. This proactive approach reduces morbidity and mortality and contributes to the overall reduction of hospital costs [6]. By addressing hemodynamic challenges promptly, healthcare providers can mitigate adverse events and optimize the recovery trajectory for patients undergoing ENT procedures.

Extubation and recovery: Two widely used anesthetic agents, lidocaine and fentanyl, have demonstrated efficacy in suppressing hemodynamic responses during the extubation phase of ENT surgeries [7]. Ensuring a seamless tracheal extubation process and facilitating proper recovery are essential to maintaining hemodynamic stability. This meticulous approach contributes to patient safety and is pivotal in minimizing complications associated with hemodynamic fluctuations. The overarching goal is to improve patient outcomes by adhering to proper monitoring practices, facilitating early detection of hemodynamic instability, and implementing timely and appropriate management strategies. The emphasis on maintaining hemodynamic stability is paramount in ensuring the well-being of patients, reducing complications, and optimizing overall surgical outcomes.

Challenges Specific to ENT Surgeries

Airway management: The intricacies of ENT surgeries often entail proximity to or intervention within the airway, presenting a substantial challenge in airway management. Patients undergoing ENT procedures are particularly prone to encountering more complex airway management issues than other surgical specialties [8]. The delicate nature of these surgeries emphasizes the critical need for specialized skills and heightened awareness in navigating the challenges associated with airway management in the ENT setting.

Postoperative complications: Unforeseen complications arising after ENT surgeries can exert significant functional and aesthetic implications for patients. The nature of postoperative complications in ENT procedures is distinctive and demands specific management principles [9]. Addressing these complications requires a nuanced understanding of the unique aspects of ENT surgery, emphasizing the importance of tailored approaches to effectively manage and mitigate the impact of postoperative issues on patient outcomes.

The global shortage of ENT services: ENT disorders face a notable lack of attention on the global health stage, contributing to a widespread shortage of ENT services. This scarcity is particularly pronounced in developing nations, posing a substantial challenge in providing adequate ear surgery services. Chronic otitis media and its complications emerge as predominant indications for ear surgery in regions grappling with this shortage, underscoring the urgency of addressing this global healthcare disparity [10]. Initiatives to bolster ENT services globally are imperative to ensure equitable access to essential care and address the specific challenges associated with ear surgeries in underserved regions.

Specific health issues: ENT surgeries encompass a spectrum of specific health concerns, addressing conditions such as recurring ear infections, frequent sinus pain or infections, deviated septum, vocal cord disorders, tonsillitis, swallowing disorders, head or neck tumors, and injuries to the face [11]. The diversity of health issues treated within the ENT domain underscores the specialized nature of these surgical interventions, necessitating tailored approaches to address each unique condition effectively.

Hemodynamic stability: Ensuring hemodynamic stability throughout ENT surgeries is paramount for safeguarding patient well-being and minimizing potential complications. A study demonstrated that the use of dexmedetomidine significantly enhanced both the quality of extubation and hemodynamic parameters in patients undergoing ENT procedures under general anesthesia [12]. The distinctive challenges ENT surgeries pose, encompassing factors such as airway management, postoperative complications, global shortages in ENT services, and the specific array of health issues addressed, underscore the importance of adhering to precise management principles. A multidisciplinary approach is essential to navigate these intricacies and achieve optimal outcomes for patients undergoing ENT interventions.

Current Strategies for Managing Hemodynamics

Controlled hypotension: Controlled hypotension, a deliberate reduction in blood pressure, is a strategic technique employed in ENT surgeries to enhance surgical visibility and minimize blood loss. Nonetheless, it is imperative to uphold hemodynamic stability throughout the intraoperative period. Achieving this delicate balance involves the precise administration of contemporary anesthetic agents and meticulous patient positioning, providing a means to effectively manage hemodynamic parameters during surgery and facilitate the controlled induction of hypotension [13]. This approach ensures optimal conditions for the surgical team while prioritizing patient safety.

Monitoring hemodynamic parameters: Rigorous monitoring of hemodynamic parameters, encompassing blood pressure, heart rate, and oxygen saturation, is indispensable in ENT surgeries. This vigilant monitoring is a crucial component of ensuring patient safety and enables the early detection of any alterations in hemodynamic status [6]. Healthcare professionals can promptly address deviations from the desired hemodynamic parameters by employing advanced monitoring techniques, contributing to enhanced patient outcomes.

Use of modern anesthetic agents: Incorporating modern anesthetic agents, exemplified by substances like dexmedetomidine, has markedly improved hemodynamic stability during ENT surgeries [5,14]. Additionally, the adoption of total intravenous anesthesia (TIVA) has gained prominence as a contemporary and practical approach in ENT procedures [13]. These advancements underscore the evolving landscape of anesthesia in ENT surgeries, emphasizing the importance of employing sophisticated agents and techniques to optimize patient care and ensure procedural success.

Preventing hemodynamic instability: Preventing postoperative hemodynamic instability is a multifaceted endeavor involving identifying and mitigating various risk factors. These contributors encompass elements related to the patient, the administration of anesthesia, and the specifics of the surgical procedure itself [6]. A proactive approach to recognizing and addressing these risk factors is pivotal, as it significantly reduces the incidence of hemodynamic instability, ultimately enhancing overall patient outcomes. By systematically managing these factors, healthcare providers can optimize the perioperative experience and minimize the risk of complications.

Fluid management: Guided intraoperative fluid management ensures optimal hemodynamic conditions, particularly in intricate procedures like free flap reconstruction in ENT surgeries [15]. This involves utilizing functional hemodynamic parameters as guides to steer fluid administration, preventing the undesirable state of hypervolemia. The tailored administration of fluids becomes imperative, especially in high-risk surgical patients, where a nuanced approach is essential to maintain fluid balance and avoid potential complications [15]. Implementing meticulous fluid management strategies contributes significantly to the safety and success of ENT surgeries, particularly in cases involving free flaps reconstruction.

Nalbuphine in pain management

Analgesic Properties of Nalbuphine

Hemodynamic impact: Nalbuphine stands out for its ability to deliver superior hemodynamic stability and analgesic effectiveness compared to other analgesics, positioning it as a preferred choice for postoperative pain relief [16]. This suggests that nalbuphine could be crucial in maintaining cardiovascular parameters within optimal ranges during the critical postoperative period, contributing to enhanced patient recovery.

Agonist/antagonist activity: As both an agonist and antagonist opioid, nalbuphine has dual mechanism-opioid-blocking properties and analgesic effects. Its analgesic potency, akin to that of morphine, underscores its efficacy in pain management across various clinical contexts [17,18]. This distinctive dual activity sets nalbuphine apart from conventional opioids, potentially offering a balanced approach to pain relief with reduced side effects.

Respiratory depression: Nalbuphine's ceiling effect for respiratory depression is noteworthy. Beyond a certain threshold, further respiratory depression is not easily induced. This characteristic, coupled with its demonstrated analgesic efficacy, positions nalbuphine as a valuable option for pain management, particularly with a diminished risk of respiratory depression compared to traditional opioids [19]. This attribute becomes especially crucial in scenarios where respiratory complications need careful consideration.

Adverse effects: Owing to its unique mixed agonist-antagonist opioid receptor activity, nalbuphine presents as an analgesic with a reduced incidence of adverse effects such as nausea, pruritus, and respiratory depression compared to morphine [19]. This suggests that nalbuphine may provide effective pain relief while minimizing common side effects associated with opioid analgesics, contributing to a more favorable patient experience during and after medical procedures.

Comparison With Other Analgesics Used in ENT Surgeries

In a clinical evaluation comparing various analgesics utilized in ENT surgeries, nalbuphine emerged as a highly effective analgesic for standard in-patient ENT procedures. The research concluded that administering nalbuphine as a single intravenous bolus during anesthesia induction ranked it among the most efficacious analgesics for routine in-patient ENT surgery, surpassing others regarding analgesic effectiveness and postoperative pain relief [20]. Notably, nalbuphine exhibited fewer adverse effects than alternative analgesics, making it a preferable option for pain management in ENT surgeries. The study underscored the significance of a judicious selection of analgesics for routine ENT surgery, considering intraoperative, recovery, and postoperative effects [20]. Moreover, a multimodal analgesia protocol, potentially incorporating nalbuphine, underwent assessment for outpatient head and neck surgical procedures, demonstrating its viability, safety, and well-received nature among patients undergoing such surgeries [21]. Nalbuphine has proven to be an effective and well-tolerated analgesic for ENT surgeries, outperforming others in analgesic efficacy while inducing fewer adverse effects. Its inclusion in a multimodal analgesia protocol has been deemed feasible and safe for outpatient head and neck surgical procedures.

Benefits and Limitations of Nalbuphine in Pain Control

Utilizing nalbuphine as a pain relief option presents distinct advantages across various dimensions. First, it has demonstrated efficacy in providing pain relief without inducing respiratory depression, making it a viable choice for addressing postoperative pain in pediatric patients [22]. Another notable attribute is its capacity to uphold hemodynamic stability, surpassing other analgesics like morphine in this crucial aspect [16]. This becomes particularly significant when maintaining stable blood pressure and heart rate is paramount for patient well-being. Patients administered with nalbuphine have exhibited superior analgesia, an improved recovery profile, and enhanced postoperative pain relief compared to those receiving morphine [16]. Additionally, the incidence of adverse effects associated with nalbuphine use is notably lower than observed with morphine, encompassing issues like hypertension, hypotension, arrhythmias, headache, dizziness, excessive drowsiness, or skin rashes [16]. Furthermore, the reduced likelihood of causing nausea and vomiting compared to morphine, leading to a more comfortable postoperative experience for patients, can be attributed to specific properties of nalbuphine. While the exact mechanisms underlying this difference require further elucidation, studies suggest that nalbuphine's unique mixed agonist-antagonist opioid receptor activity, particularly its kappa receptor agonism, may reduce gastrointestinal side effects [16]. It is essential to note, however, that despite this observed advantage, the available evidence on nalbuphine's analgesic efficacy, especially in direct comparison with other common opioids, remains a subject of ongoing investigation [22]. Additionally, the antagonist activity inherent in nalbuphine could potentially limit its analgesic effects in certain scenarios, with considerations for its interaction with spinal and epidural opioids [19]. Hence, while nalbuphine shows promise in reducing nausea and vomiting, it is imperative to acknowledge these nuances. Further research is warranted to comprehensively understand its comparative effectiveness and limitations in diverse clinical contexts.

Nalbuphine's impact on cardiovascular parameters

Blood Pressure Regulation

Systolic and diastolic effects: In a study examining the hemodynamic response to orotracheal intubation, nalbuphine was observed to prevent a significant rise in heart rate and mean arterial pressure [23]. Another study indicated that while nalbuphine had no noteworthy impact on heart rate and systolic blood pressure, it did lead to a decrease in diastolic blood pressure within 35 minutes. These reductions were statistically significant at 75 minutes and 105 minutes post-administration [24]. When compared with morphine, nalbuphine was associated with an increase in mean arterial pressure, with a higher elevation noted in the nalbuphine group at specific time points [23]. Furthermore, a study comparing nalbuphine and morphine following laryngoscopic tracheal intubation reported varied effects on mean systolic and diastolic blood pressure [25]. The influence of nalbuphine on systolic and diastolic blood pressure appears to be intricate, with studies presenting diverse findings. It is crucial to consider the specific context and patient characteristics when assessing the hemodynamic effects of nalbuphine.

Impact on mean arterial pressure: Research on nalbuphine's impact on mean arterial pressure reveals that it does not significantly affect heart rate and systolic blood pressure [24]. However, diastolic blood pressure experiences a notable decrease within 35 minutes of nalbuphine administration, and these reductions are statistically significant at 75 minutes and 105 minutes post-administration [24]. Another study found that nalbuphine effectively prevents a substantial increase in mean arterial pressure associated with laryngoscopy and orotracheal intubation [23]. In a comparative analysis with morphine, nalbuphine was identified as causing an elevation in mean arterial pressure, with a more pronounced increase observed in the nalbuphine group at specific intervals [26]. The impact of nalbuphine on mean arterial pressure is intricate, and its effects may vary depending on the specific context and characteristics of the patient.

Heart Rate Modulation

No significant effect on heart rate: In a comprehensive examination of the hemodynamic response to orotracheal intubation, nalbuphine demonstrated a lack of substantial impact on heart rate, as evidenced by findings from a study [27]. Another study corroborated this observation, which reported that nalbuphine did not induce any significant alterations in heart rate or systolic blood pressure [24].

Decrease in diastolic blood pressure: Despite its negligible influence on heart rate, nalbuphine exhibited a notable decrease in diastolic blood pressure within 35 minutes of administration. This reduction reached statistical significance at 75 and 105 minutes after administration, highlighting a distinct impact on this hemodynamic parameter [24].

Attenuation of tachycardia: Nalbuphine's efficacy in reducing tachycardia, hypertension, and cardiac workload associated with laryngoscopy and endotracheal intubation has been demonstrated in studies [27]. While nalbuphine's influence on heart rate appears minimal, its capacity to mitigate diastolic blood pressure and attenuate tachycardia becomes apparent in specific situations, reflecting its nuanced hemodynamic effects.

Vasomotor Responses

The influence of nalbuphine on vasomotor responses has been investigated in the context of laryngoscopy and tracheal intubation. In a comparative study evaluating equipotent doses of tramadol, nalbuphine, and pethidine, nalbuphine demonstrated a reduction solely in the inotropic response to airway instrumentation [28]. Additionally, another study noted that nalbuphine effectively mitigates the tachycardia, hypertension, and cardiac workload associated with laryngoscopy and endotracheal intubation [27]. However, this same study reported a non-significant decrease in heart rate and all three parameters of blood pressure (systolic, diastolic, and mean arterial pressures) within three minutes of nalbuphine administration. This observation might be attributed to its potent and predominant kappa agonistic action [27]. In another study that compared tramadol, pethidine, nalbuphine, and a placebo, the vasomotor response to laryngoscopy and tracheal intubation was found to have diminished by the time blood pressure was monitored in the post-anesthesia care unit [29]. In summary, nalbuphine's impact on vasomotor responses is intricate and may vary based on the specific context and characteristics of the patient.

Clinical Studies and Evidence on Nalbuphine in ENT Surgeries

A study comparing the efficacy of 0.2 mg/kg ketamine and 0.5 mg/kg pethidine in patients undergoing ENT surgeries revealed both drugs to be effective at the given doses. However, there is insufficient evidence to support nalbuphine as an anti-shivering agent over other drugs [30]. Nalbuphine has demonstrated its ability to provide sedation with analgesia during recovery, exhibiting a prolonged time to re-medication and a mild emetic effect. Notably, none of the analgesics studied showed evidence of respiratory depression [20]. In pediatric patients undergoing ENT surgeries, nalbuphine has been investigated in two trials comparing it with morphine. The results showed a nonsignificant lower or comparable risk ratio for moderate/severe pain at 1 hour [22]. Another study comparing morphine and nalbuphine for intraoperative and postoperative analgesia concluded that nalbuphine offers superior hemodynamic stability, analgesia, recovery profile, and postoperative pain relief compared to morphine. Furthermore, nalbuphine resulted in fewer instances of nausea and vomiting [16]. The overall effectiveness of nalbuphine in providing sedation with analgesia during recovery, coupled with its advantages in terms of hemodynamic stability, analgesia, recovery, and fewer adverse effects compared to other analgesics in ENT surgeries, has been well-established. Its particular efficacy in pediatric patients and the potential enhancement of its analgesic effects, when combined with other analgesics, further underscore its clinical utility.

Future directions

Areas for Further Research

Despite the substantial research on nalbuphine's application in ENT surgeries, areas warrant further investigation. One potential avenue for future research involves exploring the synergistic effects of nalbuphine when combined with other analgesics or anesthetics. For instance, a study in the Journal of Clinical Anesthesia revealed a synergistic impact of nalbuphine in combination with droperidol, reducing spontaneous movements in children during the induction of anesthesia with propofol [31]. Additional research could delve into the potential benefits of combining nalbuphine with other drugs to amplify its analgesic effects or mitigate adverse outcomes. Another promising area for future investigation is extending nalbuphine's application beyond ENT surgeries into other surgical specialties. While extensively studied in ENT surgeries, the potential advantages of nalbuphine in fields such as orthopedics or general surgery have yet to be thoroughly explored. Further research could evaluate the efficacy and safety of nalbuphine in these alternative surgical contexts. Finally, there is a need for more research to comprehend the long-term effects of nalbuphine use comprehensively. Despite indications that nalbuphine has fewer adverse effects compared to other analgesics, its extended impact on patient's health and recovery remains inadequately studied. Subsequent research endeavors could investigate the potential long-term consequences of nalbuphine use, especially in patients necessitating repeated surgeries or prolonged pain management.

Potential Improvements in Nalbuphine Administration

Formulation changes: Advancements in nalbuphine formulations, such as developing extended-release or slow-release versions, can enhance its bioavailability and diminish the necessity for frequent dosing. This innovation may translate into more effective pain management, potentially mitigating side effects [32].

Dosing adjustments: The optimization of nalbuphine dosing regimens presents an avenue for refining pain control and minimizing side effects. For instance, a study demonstrated the safety and efficacy of preemptive nalbuphine at a dose of 0.2−1 mg·kg in reducing postoperative visceral pain [33]. Future research endeavors could identify the optimal dosing regimen tailored to distinct patient populations and varied surgical contexts.

Co-administration with other drugs: Combining nalbuphine with other substances, such as morphine, has demonstrated efficacy in mitigating the development of morphine tolerance [34]. This collaborative approach holds promise for enhancing the overall analgesic impact while potentially diminishing the risk of addiction or dependence.

Alternative administration routes: Investigating alternative routes of administration, such as rectal or oral delivery, is a potential avenue to enhance nalbuphine bioavailability. Additionally, these alternative routes may offer patients a more convenient and accessible means of administration [32,35].

Patient-specific dosing: Tailoring nalbuphine administration to individual patient characteristics, such as weight, age, and specific response to the drug, represents a potential avenue for optimizing pain control while minimizing side effects. Implementing personalized medicine approaches in nalbuphine dosing could lead to more precise and effective pain management strategies. To fully explore the benefits of patient-specific dosing, ongoing research is essential to identify the most efficient and safe strategies for administering nalbuphine across diverse surgical contexts and patient populations [19].

Emerging Trends in Analgesia for ENT Surgeries

New developments in ENT surgeries include pre-emptive analgesia, non-opioid analgesics, and nerve stimulation therapy. A randomized controlled trial demonstrated that pre-emptive analgesia using nalbuphine and dexamethasone effectively decreased postoperative pain in pediatric otolaryngology patients [36]. Non-opioid analgesics, like NSAIDs, have exhibited the capacity to reduce postoperative vomiting and manage pain comparably to opioids [36]. Hypoglossal nerve stimulation therapy, also recognized as upper airway stimulation, emerges as a promising treatment for obstructive sleep apnea [37]. This innovative therapy involves the placement of an electrode around the hypoglossal nerve to stimulate it during sleep, thereby improving airway obstruction [37]. Furthermore, personalized medicine approaches to analgesia, including patient-specific dosing, hold promise in optimizing pain control while minimizing side effects [38]. In summary, the evolving landscape of analgesia for ENT surgeries concentrates on enhancing pain management, mitigating adverse effects, and decreasing reliance on opioids.

## Conclusions

In conclusion, the comprehensive review of the literature on nalbuphine's hemodynamic impact in ENT surgeries reveals several key findings. The studies consistently demonstrate that nalbuphine influences blood pressure and heart rate during ENT procedures, with variations observed based on dosage and patient-specific factors. While the overall assessment leans toward a positive impact on hemodynamic stability, it is essential to consider the nuances in different patient populations and the potential interaction with other analgesics or anesthetics commonly used in ENT surgeries. The practical implications for clinical practice are notable, suggesting that incorporating nalbuphine into anesthesia protocols can contribute to maintaining stable hemodynamics. However, optimal dosage and potential limitations must be addressed to ensure safe and effective implementation. This review underscores the importance of ongoing research to refine our understanding of nalbuphine's role in optimizing clinical outcomes in ENT surgeries, providing a foundation for future advancements in anesthesia management.
